# The NEDD4/FLRT2 axis regulates NSCLC cell stemness

**DOI:** 10.3389/fphar.2024.1459978

**Published:** 2024-10-09

**Authors:** Yuping Yang, Fei Yan, Ziwei Gao, Houke Li, Shengke Wen, Qi Li, Jiayuan Li, Na Huang, Wei Zhao

**Affiliations:** ^1^ Department of Respiratory and Critical Care Medicine, School of Clinical Medicine and The First Affiliated Hospital of Chengdu Medical College, School of Clinical Medicine, of Chengdu Medical College, Chengdu, China; ^2^ Key Laboratory of Geriatic Respiratory Diseases of Sichuan Higher Education Institutes, School of Clinical Medicine and The First Affiliated Hospital of Chengdu Medical College, Chengdu, China; ^3^ Department of Medical Oncology, Jiangsu Cancer Hospital and Jiangsu Institute of Cancer Research and The Affiliated Cancer Hospital of Nanjing Medical University, Nanjing, China; ^4^ School of Laboratory Medicine, Chengdu Medical College, Chengdu, Sichuan, China; ^5^ School of Clinical Medicine, Chengdu Medical College, Chengdu, China; ^6^ Department of Clinical Laboratory, School of Clinical Medicine and The First Affiliated Hospital of Chengdu Medical College, Chengdu, China

**Keywords:** FLRT2, Nedd4, non-small cell lung cancer, stemness, degradation

## Abstract

**Introduction:**

Lung cancer is the leading cause of cancer-related death worldwide. The treatment for lung cancer, particularly for non-small cell lung cancer (NSCLC), remains a clinical challenge. Cancer stem cells are vital for lung cancer development. This study aimed to determine the influence of the neuronally expressed developmentally downregulated 4-fibronectin leucine-rich transmembrane 2 (NEDD4-FLRT2) axis on cancer cell stemness in NSCLC.

**Methods:**

FLRT2 expression in NSCLC tissues and stem cells was investigated using western blot and RT-qPCR. The sphere formation assay and the abundance of stemness markers were employed to confirm the stemness of NSCLC stem cells. The CCK-8, colony formation, and Trans-well assays, as well as flow cytometry, were used to determine NSCLC stem cell growth, metastasis, and apoptosis, respectively. The Co-IP assay was used to confirm the binding between NEDD4 and FLRT2. Xenograft tumor mouse models were used to investigate tumorigenesis *in vivo*.

**Results:**

Here, we reported that FLRT2 expression was reduced in NSCLC tissues, cells, and NSCLC stem cells. FLRT2 upregulation inhibited NSCLC stem cell proliferation, sphere formation, and drug resistance and promoted drug-resistant cell apoptosis. Furthermore, FLRT2 overexpression demonstrated antitumor effects in a xenograft tumor mouse model. Mechanically, FLRT2 was ubiquitinated and degraded by E3 ligase NEDD4. NEDD4 overexpression significantly abolished the inhibitory effects of FLRT2 on NSCLC stemness, as evidenced by in vitro and in vivo experiments.

**Discussion:**

This study revealed that FLRT2 acted as a tumor suppressor by inhibiting cancer cell stemness in NSCLC. NEDD4 promoted ubiquitination degradation of FLRT2 protein. NEDD4 counteracted the inhibitory effects of FLRT2 on NSCLC stem cell tumorigenesis.

## Introduction

Lung cancer is a major global concern owing to its high incidence and mortality rates (Yu et al., 2021; Leiter et al., 2023). According to the recent global cancer statistics, lung cancer is the second most common and deadliest tumor worldwide (Bray et al., 2024). Non-small cell lung cancer (NSCLC) is the most common histological type, accounting for 80%–85% of all lung cancer cases (Duan et al., 2022). Despite recent advances in cancer therapy, many treatments available for patients with lung cancer remain unsatisfatory. One of the most common reasons for the treatment failure is the presence of lung cancer stem cells (CSCs) (Fakiruddin et al., 2021).

The CSC hypothesis has received a great deal of attention in the last 2 decades from both a tumor conceptual and therapeutic standpoint (Heng et al., 2019). CSC, a highly tumorigenic subpopulation, is thought to be responsible for tumor growth, drug resistance, metastasis, and recurrence (Cruz Walma et al., 2022). Evidence suggests that CSCs are vital for tumorigenesis and NSCLC progression (Bertolini and Roato, 2022; Panda and Biswal, 2022; Yu et al., 2022). CD44, OCT4, SOX2, and Nanog have been identified as core factors that maintain stem cell properties such as self-renewal, tumorigenesis, and metastasis (Singh et al., 2012; Alnasser, 2022; Lu Y. et al., 2022). Another stem cell surface marker, CD133 was found as a prognostic marker for NSCLC. The CD133 high expression is also associated with shorter overall and recurrence-free survival in patients with NSCLC (Wu et al., 2014). OCT4 and SOX2 induced by HIF1 and HIF2 were upregulated in the CD133 promoter in NSCLC cells under hypoxic conditions. OCT4 and Nanog overexpression in NSCLC cells induced stem cell properties (Iida et al., 2012; Singh and Chellappan, 2014). Therefore, it is essential to explore the potential mechanisms underlying NSCLC stemness.

Fibronectin leucine-rich transmembrane 2 (FLRT2) is a member of the FLRT family, characterized by a type III fibronectin domain and conserved 10 leucine-rich repeats domains (Bae et al., 2017). Some studies have reported FLRT2’s role in tumor development. For example, FLRT2 is widely expressed in abnormal blood vessels in advanced colorectal cancer, and its expression is negatively correlated with long-term survival (Ando et al., 2022). Contrarily, FLRT2 downregulation was observed in colorectal cancer and breast cancer, and FLRT2 inhibited colorectal cancer (Guo et al., 2020) and breast cancer (Bae et al., 2017) progression. Methylation of FLRT2 has been linked to prostate cancer progression (Wu et al., 2016). Recently, FLRT2 was reported to suppress bladder cancer progression by inducing ferroptosis (Jiang et al., 2024). However, its role in stemness and tumorigenesis of NSCLC remains unknown.

Our preliminary study depicted that the neuronally expressed developmentally downregulated 4 (NEDD4) may serve as an E3 ligase of FLRT2. NEDD4 is widely expressed in mammalian tissues and can mediate protein ubiquitination, proteasome degradation, receptor-mediated endocytosis, and protein transport (Lu X. et al., 2022). NEDD4 is overexpressed in various human malignant tumors, including gastric, colorectal, prostate, lung, liver, and breast cancers (Cao et al., 2023; He et al., 2024). Emgering data shown NEDD4 promotes cancer progression through ubiquitination-depedent degradation. NEDD4-dependent degradation accelerates breast cancer cells metastasis (Luo et al., 2022), and enhances colorectal cancer cells chemoresistance to 5-fluorouracil (Anand et al., 2023). NEDD4 promotes lung cancer cell migration, drug resistance, and metabolism reprogramming (Zhong et al.; Sun et al., 2017; Shao et al., 2018; Sun A. Q. et al., 2021). These findings indicate that NEDD4 is essential for cancer and stem cells. However, the function of NEDD4 in NSCLC stem cells has not yet been elucidated. In this study, we explored the effects of the NEDD4-FLRT2 axis on the stemness of NSCLC cells in NSCLC development and its mechanisms.

## Materials and methods

### Clinical samples

The clinical tissue samples were obtained from 50 patients diagnosed with NSCLC who underwent surgery at the First Affiliated Hospital of Chengdu Medical College from September 2018 to October 2020. Written informed consents were obtained from all patients before enrollment in the study. The study procedures were approved by the ethics committee of the First Affiliated Hospital of Chengdu Medical College.

### Cell culture, Cell transfection and stem-like NSCLC cell isolation

Lung cancer cell lines A549, H1975, NCI-H270, PC-9, and SPCA-1 were cultured in DMEM (Gibco, USA), supplemented with 10% fetal bovine serum (FBS; Gibco, USA) and 1% antibiotics (Thermo, USA). NSCLC stem cells were isolated from A549 and H1975 cell lines following our lab protocol. For cells transfection was described in our previous report (Zhao et al., 2024). Briefly, the lentiviral supernatant was used to transduce cells under the instruction. Finally, the infected cells were selected by puromycin (Thermo Fisher Scientific, Cat. No.: A1113802) for 7 days, and the expression was checked by RNA extraction and quantitative real-time PCR (qRT-PCR).

Briefly, A549 and H1975 cells were seeded at a concentration of 2 × 10^3^ cells/well in 6-well plates and cultured in DMEM supplemented with FBS for 10 days. The suspended cells were then isolated and cultured in serum-free DMEM for another 21 days until the tumor spheres formed. Subsequently, we assessed the stemness of stem-like NSCLC cells using a sphere formation assay and expression of stem cell markers. All cells were maintained at 37°C in the incubator with 5% CO_2_.

### Bioinformatic analysis

The survival distribution of NSLC patients were analyzed by online Kaplan–Meier software (http://kmplot.com/analysis/index.php?p=service&cancer=lung) based on the data from a previous report (Gyorffy, 2024) and gene expression profiling interactive analysis (GEPIA2, http://gepia2.cancer-pku.cn/#index) (Li et al., 2021). FLRT2 mRNA expression in clinical specimens and different cells were analyzed in GEO database (https://www.ncbi.nlm.nih.gov/geo/) and GEPIA2 (http://gepia2.cancer-pku.cn/#index). The correlation of cisplatin half maximal inhibitory concentration (IC_50_, https://www.aclbi.com/static/index.html#/drug_allergy)/stemness (https://www.aclbi.com/static/index.html#/stem_cells) scores with FLRT2 expression were analyzed by R package which were implemented by R foundation for statistical computing (2020) online (version 4.0.3). Moreover, UbiBrowser (http://ubibrowser.ncpsb.org.cn) was utilized to systematically identify the potential E3 ligase of FLRT2 (Li et al., 2017).

### RT-qPCR

Total RNA was isolated from tissues and cells, and cDNA was generated. RT-qPCR was performed using SYBR Green Mix (TOYOBO, Japan) with primers for FLRT2, OCT4, CD44, CD133, NEDD4, Cyclin A1, PCNA, E-cadherin, N-cadherin, Bax, and Bcl-2. The primers used are listed in Table 1.

**TABLE 1:** Primers of RT-qPCR.

Gene name	Forward (5’-3’)	Reverse (5’-3’)
FLRT2	5’-TCT GAG CAG CCT CCA GAC C-3’	5’-CGG AGC TCC TCG GAT TTG G-3’
OCT4	5’-CCT TCG CAA GCC CTC ATT TC-3’	5’-TAG CCA GGT CCG AGG ATC AA-3’
CD44	5’-CAG CTC ATA CCA GCC ATC CA-3’	5’-GCT TGA TGA CCT CGT CCC AT-3’
CD133	5’-CCC CGC AGG AGT GAA TCT TT-3’	5’-GAA GGA CTC GTT GCT GGT GA-3’
NEDD4	5’-AAG CGT TCG GAA ATG GCA AC-3’	5’-GCA AGG CCT ATT CCG GCT AT-3’
Cyclin A1	5’-CAC CCT GCT CGT CAC TTG G-3’	5’-ACG GGC TGC TGC TGG AA-3’
PCNA	5’-GAG CTC TTC CCT TAC GCA AGT CT-3’	5’-CAG GCG GGA AGG AGG AAA GT-3’
E-cadherin	5’-GCT GGA CCG AGA GAG TTT CC-3’	5’-CGA CGT TAG CCT CGT TCT CA-3’
N-cadherin	5’-AGG CTT CTG GTG AAA TCG CA-3’	5’-AAA TCT GCA GGC TCA CTG CT-3’
Bax	5’-GAC GGG TCC GGG GAG-3’	5’-CTC GAT CCT GGA TGA AAC CCT G-3’
Bcl-2	5’-GAT AAC GGA GGC TGG GAT GC-3’	5’-TCA CTT GTG GCC CAG ATA GG-3’
GAPDH	5′-CAC CCA CTC CTC CAC CTT TG-3′	5′-CCA CCA CCC TGT TGC TGT AG-3′

### Western blot analysis

NSCLC cells were lysed in protein lysis buffer (RIPA buffer supplemented with 1% proteinase inhibitor). Proteins were separated by sodium dodecyl sulfate-polyacrylamide gel electrophoresis (SDS-PAGE) and transferred to the NC membranes. The NC membranes were incubated overnight at 4°C with primary antibodies for FLRT2 (Proteintech, China), OCT4 (CST, USA), CD44 (Abcam, United Kingdom), CD133 (Invitrogen, USA), E-cadherin (Bioworld, China), N-cadherin (Invitrogen, USA), Bax (Proteintech, China), Bcl-2 (Proteintech, China), and NEDD4 (Abcam, United Kingdom), respectively. The membranes were then incubated with secondary antibodies (ZSJQ, China) for 50 min at room temperature. Finally, the protein bands were detected using an ECL Assay Kit (Beyotime, China).

### Sphere formation assay

NSCLC cells were seeded in 6-well plates at a density of 750 cells/well in DMEM supplemented with 5 mg/L heparin (Bio-techne, China), 20 μg/L hEGF (Sigma, USA), 2% B27 (Sigma, USA), and 10 μg/L bFGF (Sigma, USA). After 12-day culture, the number and size of spheroids were observed, and spheres of appropriate size (diameter >60 μm) and appearance were measured under a microscope as previously reported (Zhou et al., 2015; Herreros-Pomares et al., 2019).

### Cell counting kit-8 (CCK-8) assay

Cell viability was evaluated using a CCK-8 kit (MCE, China). 1,000 cells were seeded in each well of a 96-well plate. CCK-8 solution was added to each well and maintained for 2 h at 37°C. Finally, the optical density of each well was measured at 450 nm.

### Colony formation assay

The cells were seeded at a density of 750 cells/well in a 6-well plate and cultured at 37°C. After 10 days, the cells were washed and fixed in 4% PFA (Beyotime, China), followed by staining with LCV (Beyotime, China). After washing and drying, the colonies were counted under a microscope.

### Transwell assay

Briefly, 2 × 10^5^ cells were seeded into the upper chamber and maintained in serum-free DMEM. DMEM supplemented with 10% FBS was placed in the lower chamber. The cells were cultured for 24 h and fixed with 4% PFA for 15 min at room temperature. Cells that migrated to the lower chamber were visualized under a microscope.

### Cell apoptosis assay

The Annexin V-FITC kit (MCE, China) was used to detect cell apoptosis following the manufacturer’s instructions. Briefly, cells were suspended in a binding buffer, then incubated for 10 min at 4°C with 5 μL Annexin V-FITC. Then, 10 µL of PI solution was added and incubated at 4°C for another 5 min. Flow cytometry was used to examine the apoptotic cells.

### Co-IP assay

Cells were lysed using lysis buffer, and total proteins were collected. The magnetic beads (MCE, China) were co-incubated with the antibody against FLRT2 (Proteintech, China) for half an hour at room temperature. The beads were then separated and co-cultured with the proteins for 1 h at room temperature. Finally, the magnetic beads were separated, and the target protein was detected by Western blotting.

### Luciferase screening

The potential downstream signaling pathway(s) regulated by NEDD4/FLRT2 were screened using the Cignal Reporter Assay Kit (Qiagen, Shanghai, China, Cat. No. 336821) as in our previous reports (Wu et al., 2018; Yang et al., 2021). Briefly, NSCLC cells were seeded into a 96-well plate at 1,000/well concentration. Cells were transfected with plasmids in the kit and harvested for luciferase activity test according to the manufacturer’s instructions.

### Xenograft tumor mouse models

Xenograft tumor mouse models were generated as described in our previous reports (Li et al., 2020; Zhuang et al., 2024). Briefly, the male nude mice aged 4–5 weeks were bought from GemPharmatech (Chengdu, China) and housed in an SPF room for a 7-day adaptive phase. All animal experiments were approved by the Research Ethics Committee of the Chengdu Medical College. The stem-like NSCLC cells were collected, and density was adjusted to 1 × 10^7^ cells/mL. In the hind legs of each mouse, 100 μL of 1 × 10^6^ cells was injected subcutaneously. Tumor size was measured every 3 days, and on the 24th day, the tumors were excised and weighed.

### Statistical analysis

GraphPad Prism software was used to analyze experimental data. Figures are presented as mean ± standard error of the mean (SEM). One-way analysis of variance or Student’s t-test was used to compare groups.

## Results

### Decreased FLRT2 was correlated with poor clinical outcome and high NSCLC cell stemness status

**FIGURE 1:**
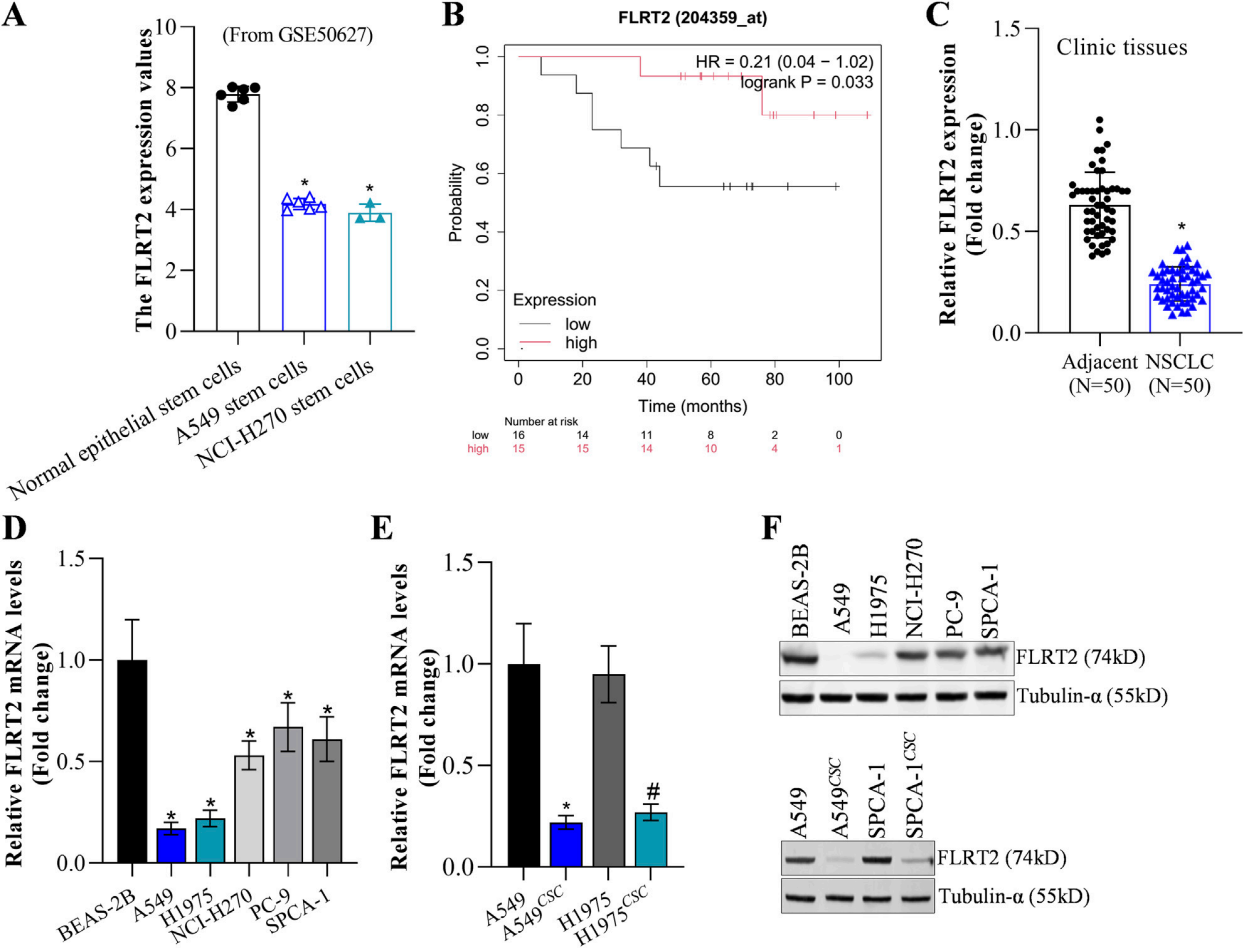
FLRT2 expression in NSCLC and NSCLC^CSC^. **(A)** FLRT2 expression in lung cancer stem cells was analyzed in the GEO database. **(B)** FLRT2 level was involved in NSCLC prognosis. **(C)** RT-qPCR analysis compared the mRNA level of FLRT2 in NSCLC and normal tissues. **(D)** mRNA abundance of FLRT2 was examined in normal lung epithelial and lung cancer cells. **(E)** mRNA level of FLRT2 was examined in NSCLC and NSCLC^CSC^ cells. * represented comparison with the A549 group, # represented comparison with the H1975 group. **(F)** FLRT2 protein abundance was examined in normal lung epithelial cells, lung cancer cells, and NSCLC^CSC^. All data are expressed as mean ± SEM of three independent experiments. *p < 0.05, **p < 0.01, and ***p < 0.001 assessed via a two-tailed t-test; ns: non-significant.

FLRT2 mRNA level had no significant difference between NCLC tumors and normal tissues based on the GEPIA2 database (Supplementary Figure S1A). However, the GEO database (GSE50627) revealed that A549 and NCI-H270 stem cells exhibited lower FLRT2 expression (Figure 1A). Bioinformatics analysis depicted that FLRT2 expression was negatively correlated with NSCLC stemness (Supplementary Figure S1B). Based on the online Kaplan-Meier plotter (Győrffy, 2024), patients with relatively higher FLRT2 expression displayed longer survival durations (Figure 1B), implying that FLRT2 was positively associated with lung cancer prognosis. Our collected tumors revealed that NSCLC tissues expressed less FLRT2 than the adjacent normal lung tissues (Figure 1C). FLRT2 abundance was detected in normal lung epithelial cell line BEAS-2B and lung cancer cell lines A549, H1975, NCI-H270, PC-9, and SPCA-1. FLRT2 exhibited a significant reduction in all lung cancer cell lines from mRNA and protein levels compared to BEAS-2B cells (Figures 1D–F). We then compared FLRT2 levels in NSCLC cells and stem cells isolated from patients with NSCLC. FLRT2 expression was extremely low in A549 and H1975 stem cells (Figures 1E, F). These findings suggested that FLRT2 may play a suppressing role in the stemness of NSCLC.

### FLRT2 inhibited the stemness of NSCLC cells

**FIGURE 2:**
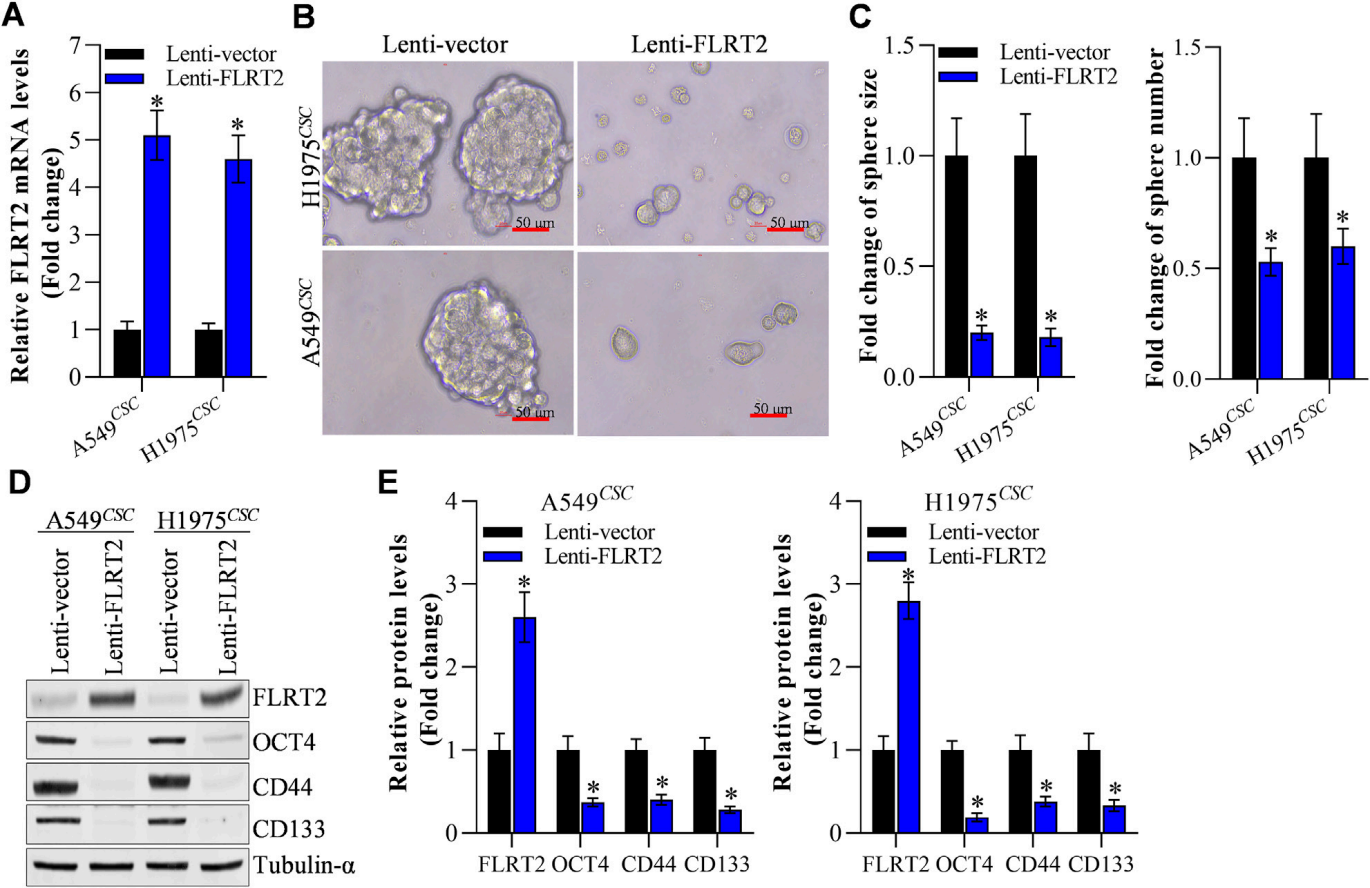
FLRT2 overexpression inhibits the stemness of NSCLC cells. **(A)** The efficacy of FLRT2 overexpression was measured after transfection for 48 h. **(B)** Evaluation of tumor sphere formation after FLRT2 upregulation. **(C)** Statistical data revealed the size and number of the spheres in the pcDNA 3.1 and FLRT2 groups. **(D)** Change in the protein level of stem cell markers after FLRT2 overexpression. **(E)** Change in mRNA level of stem cell markers after FLRT2 overexpression. All data are expressed as mean ± SEM of three independent experiments. *p < 0.05, **p < 0.01, and ***p < 0.001 assessed via a two-tailed t-test; ns: non-significant.

Based on our previous reports (Li et al., 2020; Zhuang et al., 2024), A549 and H1975 cells with stable FLRT2 overexpression were constructed. Figure 2A displays that FLRT2 was successfully overexpressed in A549 and H1975 stem cells. Tumor sphere-formation data revealed that cells in the FLRT2 group had significantly lower sphere-forming ability than cells in the control group (Figures 2B, C). FLRT2 upregulation decreased the protein and mRNA levels of stem cell markers OCT4, CD44, and CD133 in A549 and H1975 stem cells (Figures 2D, E). These findings implied that FLRT2 overexpression limited stem cell marker expression and inhibited the stemness of NSCLC cells to form tumor spheres.

### FLRT2 overexpression deterred NSCLC cell proliferation and drug resistance

**FIGURE 3:**
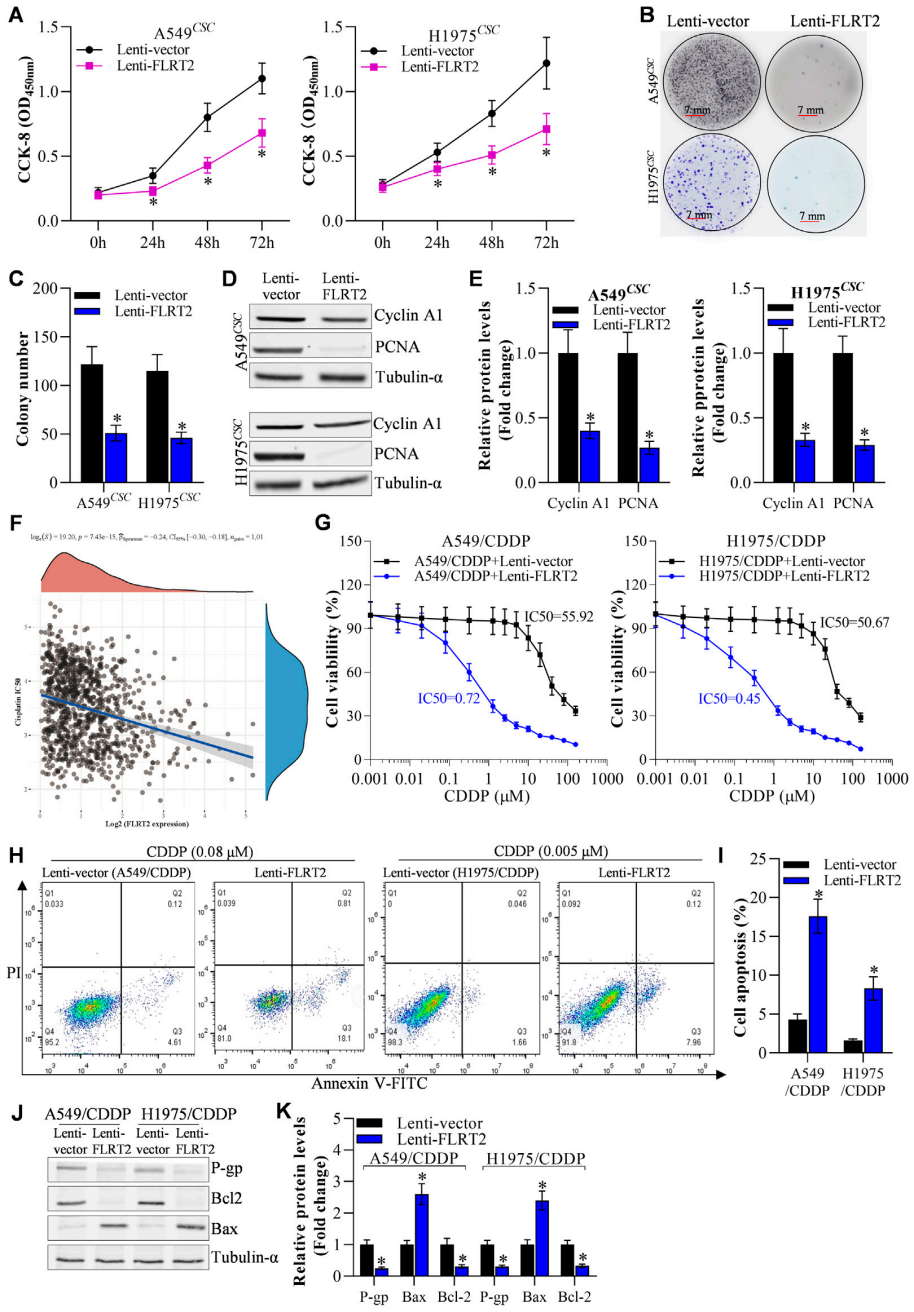
Overexpression of FLRT2 inhibits NSCLCCSC proliferation and drug resistance. **(A)** Viability of NSCLCCSC detected by CCK-8 assay. **(B)** Proliferation of NSCLCCSC detected by colony formation assay. **(C)** Changes in colony number. **(D, E)** Western blotting detected proliferation markers. **(F)** Bioinformation analyzed FLRET2 correlation with NSCLC cells cisplatin resistance. **(G)** CCK-8, flow cytometry **(H, I)** and western blotting **(J, K)** detecte NSCLC cisplatin resistance cells (A549/CDDP and H1975/CDDP) IC50, cell death and cell drug resistance-related proteins to cisplatin when knockdown FLRT2, respectively. All data are expressed as mean ± SEM of three independent experiments. *p < 0.05, **p < 0.01, and ***p < 0.001 assessed via a two-tailed t-test; ns: non-significant.

To determine the role of FLRT2 upregulation in NSCLC stem cell growth, we first examined the viability of A549 and H1975 stem cells. CCK-8 assay revealed that FLRT2 overexpression reduced the viability of A549 and H1975 stem cells at 24, 48, and 72 h (Figure 3A). The colony formation assay was used to assess NSCLC stem cell proliferation. FLRT2 inhibited NSCLC stem cell proliferation, as depicted in Figures 3B, C. FLRT2 upregulation also reduced the levels of proliferation markers (Cyclin A1 and PCNA) (Figures 3D, E). As the stemness of cancer cells is a major cause of drug resistance (Shen, 2022), both bioinformatics analysis (Figure 3F) and IC_50_ value detection (Figure 3G) exhibited that FLRT2 abrogates NSCLC cells cisplatin resistance. Consistent with this, FLRT2 also promoted cisplatin-resistant cell (A549/CDDP and H1975/CDDP) apoptosis (Figures 3H, I), with the downregulation of P-gp/Bcl2 and upregulation of Bax (Figures 3J, K). These findings suggested that FLRT2 overexpression is responsible for inhibiting NSCLC stem cell proliferation and drug resistance.

### Ubiquitination degradation of FLRT2 protein was mediated by NEDD4

**FIGURE 4:**
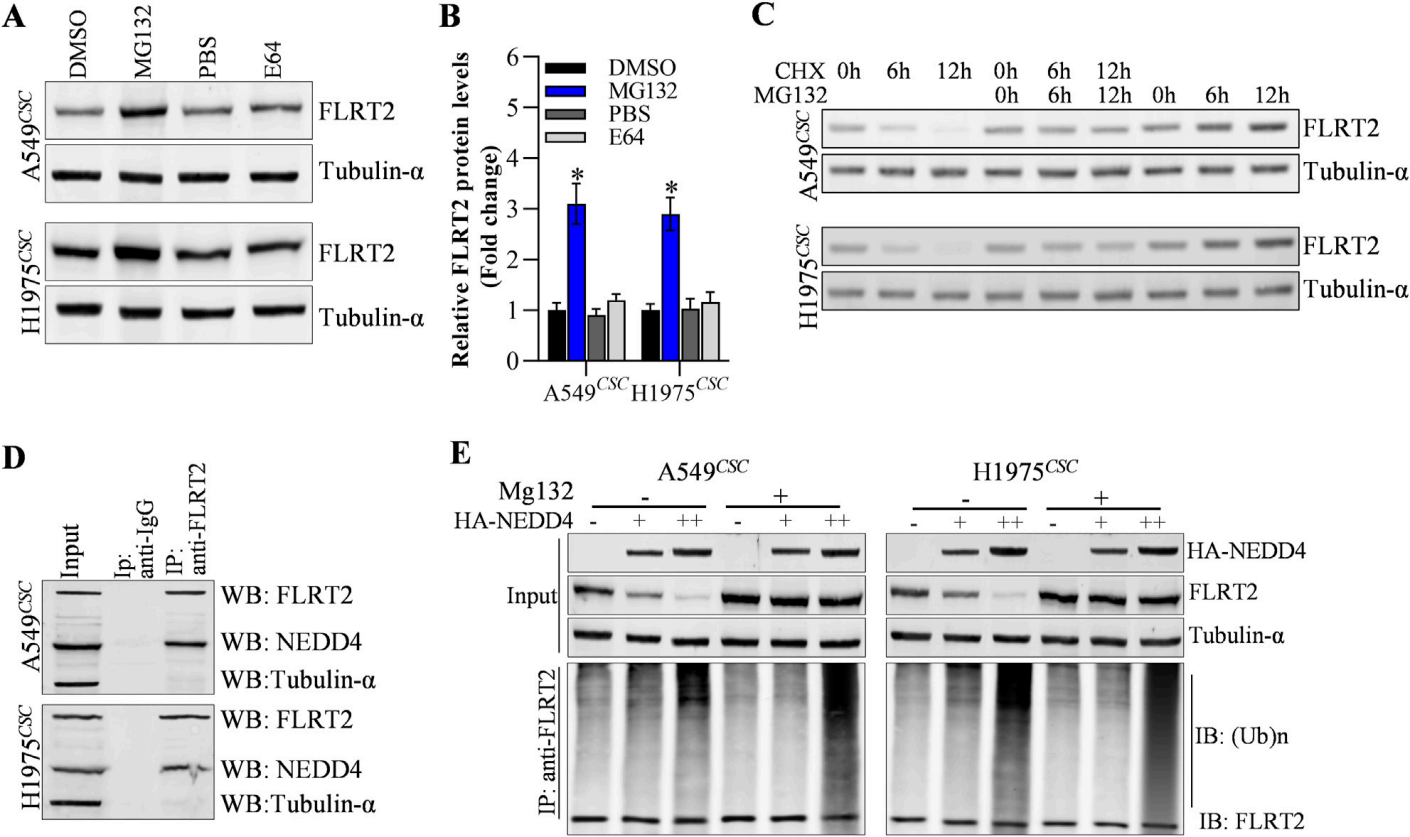
NEDD4 mediates the ubiquitination of FLRT2. **(A–C)** FLRT2 is regulated by proteasome degradation. Cells were incubated with MG132 or E64, CHX at indicated concentration for protein level detection by Western blot. **(D)** Co-IP depicted the interaction between NEDD4 and FLRT2. **(E)** NEDD4 regulated the ubiquitination degradation of FLRT2. All data are expressed as mean ± SEM of three independent experiments. *p < 0.05, **p < 0.01, and ***p < 0.001 assessed via a two-tailed t-test; ns: non-significant.

Next, we explored the potential mechanisms underlying FLRT2 protein expression. In this study, we discovered that cells treated with MG132 (proteasome degradation pathway inhibitor) had an increased abundance of FLRT2, whereas E64 (lysosome degradation pathway inhibitor) had no effect on FLRT2 expression in A549^CSC^ and H1975^CSC^ cells (Figures 4A, B). Moreover, cycloheximide (CHX, protein synthesis inhibitor) obviously decreased FLRT2 protein levels at a time-dependent manner, and MG132 significantly inhibited CHX-mediated downregulation of FLRT1 protein (Figure 4C). These findings suggest that ubiquitination-mediated proteasomal degradation plays a vital role in FLRT2 regulation. Therefore, we concentrated on identifying E3 ligases that affected FLRT2 ubiquitination. According to the prediction from Unibrowser database, NEDD4 is the promosing E3 ligase for the ubiquitination of FLRT2 (Supplementary Figure S2). Therefore, we examined the interaction between NEDD4 and FLRT2. Co-IP results revealed that NEDD4 bound to FLRT2 in A549^CSC^ and H1975^CSC^ cells (Figure 4D) and that NEDD4 could induce ubiquitination and degradation of FLRT2 protein (Figure 4E).

### Decreased NEDD4 abrogated the NSCLC stemness and benefited clinical outcome

**FIGURE 5:**
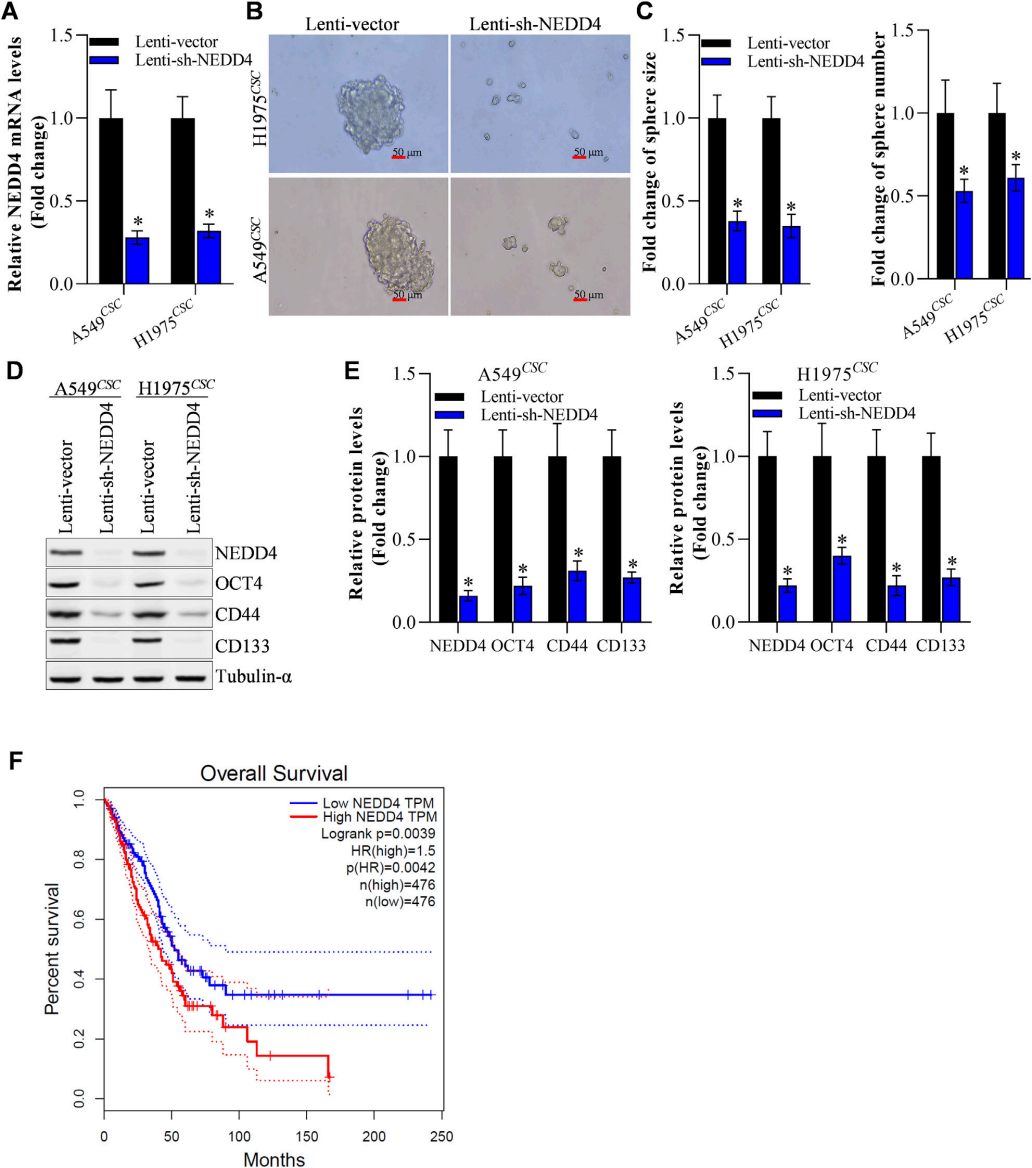
NEDD4 knockdown restrains the stemness of NSCLCCSC. **(A)** Efficiency of NEDD4 knockdown. **(B, C)** Knockdown of NEDD4 restrained tumor sphere formation of NSCLCCSC. **(D)** Change in the protein level of stem cellmarkers after NEDD4 knockdown. **(E)** Change in mRNA level of stem cellmarkers after NEDD4 knockdown. **(F)** Survival rate difference between NEDD4-high and NEDD4-low NSCLC patients group. All data are expressed as mean ± SEM of three independent experiments. *p < 0.05, **p < 0.01, and ***p < 0.001 assessed via a twotailed t-test; ns: non-significant.

Furthermore, we explored whether NEDD4 influenced NSCLC stemness. RT-qPCR results revealed that the NEDD4 mRNA level was reduced in A549^CSC^ and H1975^CSC^ cells (Figure 5A). NEDD4 knockdown weakened A549^CSC^ and H1975^CSC^ tumor sphere formation (size and number) (Figures 5B, C). After NEDD4 downregulation, the expression of stem cell markers, including OCT4, CD44, and CD133, decreased at the protein and mRNA levels (Figures 5D, E). According to the GEPIA2 database, patients with NSCLC tumors had a better clinical survival rate in low NEDD4 group than those in the NEDD4-high group (Figure 5F). These data suggest that NEDD4 functions as an oncogene by increasing NSCLC cell stemness.

### NEDD4 knockdown weakened the tumorigenesis of NSCLCs *in vivo*

**FIGURE 6:**
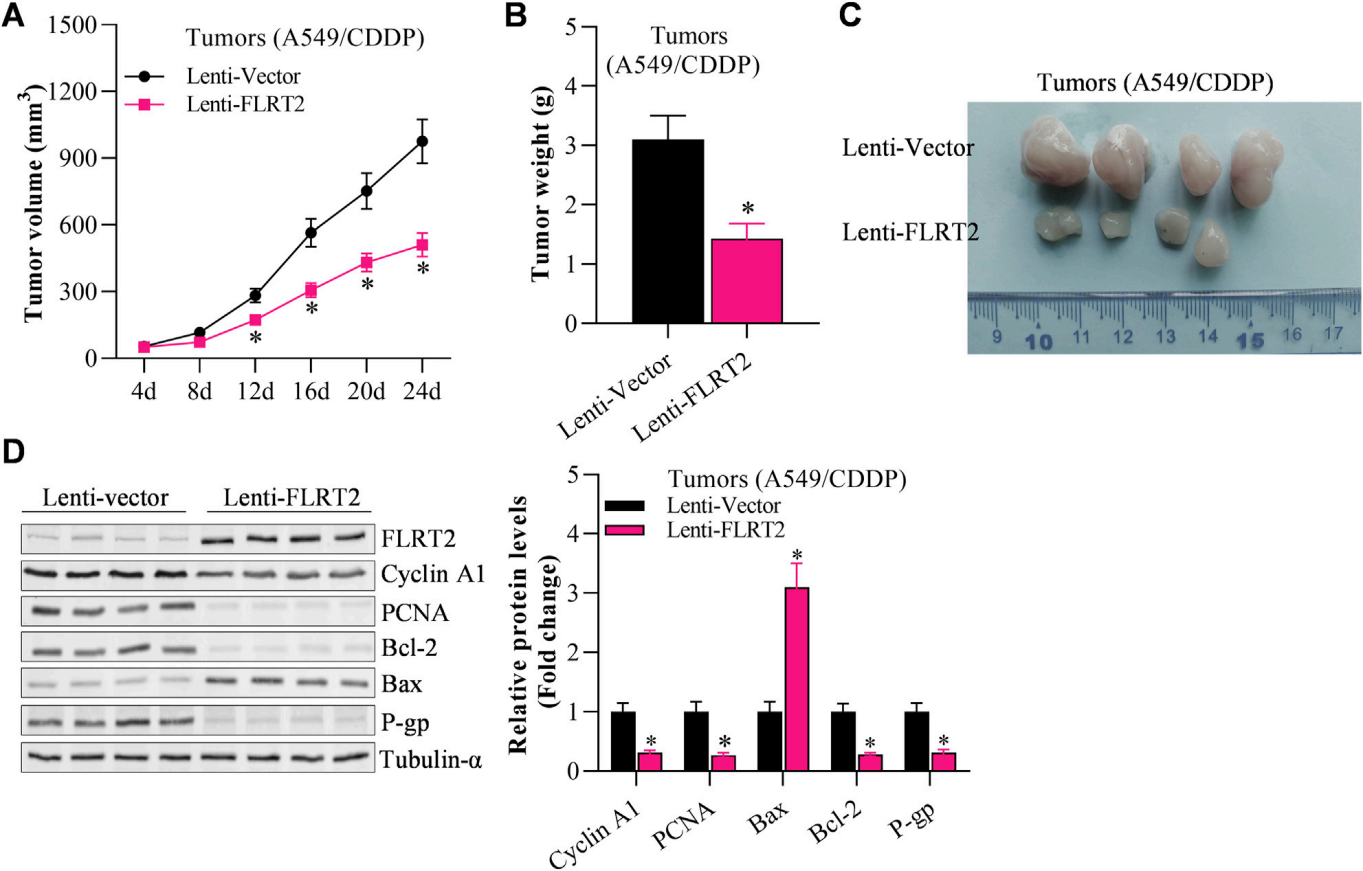
NEDD4 knockdown restrains the tumorigenesis of NSCLC^CSC^. **(A)** Tumor volume of A549^CSC^-injected mice. **(B)** Tumor weight of A549 ^CSC^-injected mice. **(C)** Tumors excised from A549^CSC^-injected mice. **(D)** Abundance of proliferation-, EMT-, and apoptosis-associated genes in the tumor tissues of A549^CSC^-injected mice. All data are expressed as mean ± SEM of three independent experiments. *p < 0.05, **p < 0.01, and ***p < 0.001 assessed via a two-tailed t-test; ns: non-significant.

The above findings demonstrated NEDD4’s pro-stemness functions by inhibiting sphere formation in NSCLC stem cells. Therefore, we hypothesized that NEDD4 would inhibit tumor growth *in vivo*. To test our hypothesis, we subcutaneously injected A549^CSC^ cells into nude mice using a xenograft tumor model. Tumor formation and progression were monitored for 24 days. The results depicted that the tumor volume and weight were reduced in the lenti-sh-NEDD4 group compared to the lenti-vector group (Figures 6A–C). Furthermore, we examined the proliferation, metastasis, stemness and apoptosis marker expression in tumor tissues (Figure 6D). Immunoblot revealed that the proliferation, drug resistance and stemness biomarker proteins were significantly downregulated in the lenti-sh-NEDD4 group. Regarding EMT markers, NEDD4 knockdown increased E-cadherin and decreased N-cadherin levels. Furthermore, the apoptotic activator Bax was upregulated, while the apoptotic inhibitor Bcl-2 was downregulated in the lenti-sh-NEDD4 group (Figure 6D). These findings exhibited that NEDD4 knockdown inhibited tumorigenesis of NSCLC stem cells *in vivo*.

### NEDD4 significantly counteracted the inhibitory effects of FLRT2 on the tumorigenesis of NSCLC stem cells

**FIGURE 7:**
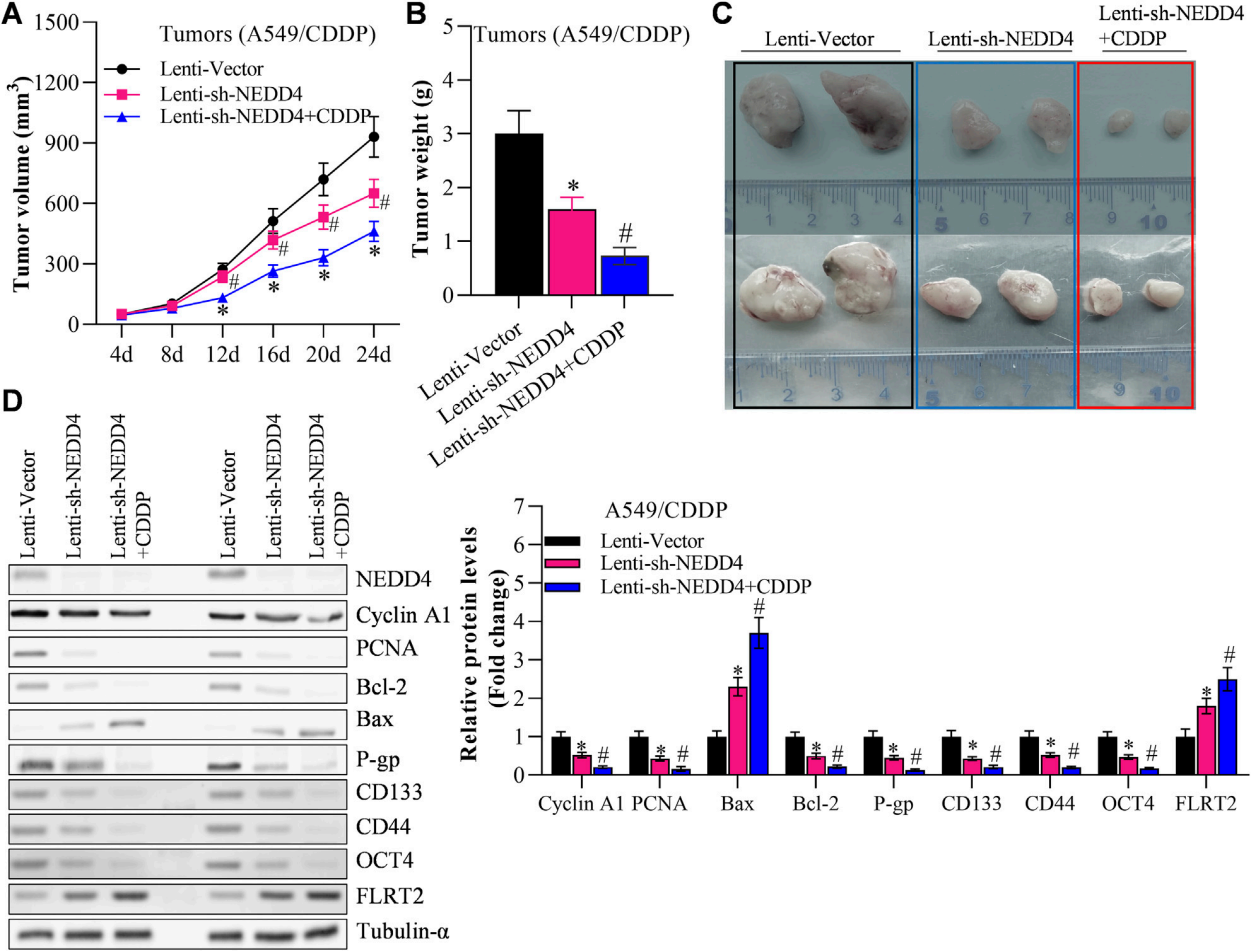
NEDD4 overexpression counteracts the inhibitory effects of FLRT2 on tumorigenesis of A549^CSC^. **(A)** Tumor volume increased in the NEDD4 overexpression group compared with the FLRT2 knockdown group. **(B)** Tumor weight increased in the NEDD4 overexpression group compared with the FLRT2 knockdown group. **(C)** Tumors were excised from A549^CSC^-injected mice in the indicated three groups. **(D)** NEDD4 overexpression neutralized the effects of FLRT2 downregulation on the abundance of proliferation-, EMT-, and apoptosis-associated genes. All data are expressed as mean ± SEM of three independent experiments. *p < 0.05, **p < 0.01, and ***p < 0.001 assessed via a two-tailed t-test; ns: non-significant.

Finally, we investigated the effects of NEDD4/FLRT2 interaction on NSCLC stemness in *in vivo* models. Based on our data, we hypothesized that NEDD4 blocks the influence of FLRT2 on NSCLC stemness. To test this hypothesis, we used a xenograft mouse model. When A549^CSC^ cells were transfected with lenti-FLRT2, tumor volume, and weight were significantly reduced, whereas NEDD4 overexpression blocked the inhibitory effects of lenti-FLRT2 on tumor growth (Figures 7A–C). Furthermore, NEDD4 reversed the reduction of FLRT2 on the expressions of stemness markers as well as proliferation and metastasis related marker in stem cells (Figure 7D), as well as stemness-related signal pathways, such as HIF-1α, NF-kB and TCF/LEF when screened by Cignal Reporter Assay Kits (Supplementary Figure S3). Our findings suggested that the NEDD4/FLRT2 axis contributes to NSCLC stemness.

## Discussion

Among all cancers, lung cancer is the leading cause of cancer-related death (Wathoni et al., 2022). Patients with lung cancer have a low survival rate and are prone to recurrence after surgery or radiotherapy, which is thought to be due to the presence of CSCs (Lai et al., 2018). Although CSCs constitute a small percentage of tumor cells, this cell subpopulation is the primary cause of cancer development. Therefore, targeting CSCs is the key in tumor treatment. In this study, we investigated the regulatory role of FLRT2 in cancer cell stemness in NSCLC and the underlying mechanism. This study reported that FLRT2 is downregulated in NSCLC, particularly in NSCLC stem cells, and FLRT2 overexpression effectively suppressed the growth and metastasis of NSCLC stem cells. NEDD4 mitigated FLRT2 effects on NSCLC stemness by mediating FLRT2 ubiquitination and degradation. NEDD4 increases cancer stemness, FLRT2 abrogates the NEDD4-induced NSCLC progression. These data hints FLRT2 might serve as a therapeutic target and the potential biological barriers to developing NEDD4 inhibitors.

Yoshiaki Kubota et al. reported that FLRT2 is overexpressed in abnormalized vessels of CRC, and its overexpression is associated with a shorter survival time (Ando et al., 2022). Contrarily, FLRT2 was downregulated in CRC due to the hypermethylation of its promoter, and the FLRT2 overexpression could impede the proliferation and metastasis of CRC cells (Guo et al., 2020). However, FLRT2 is a tumor suppressor in the liver (Song et al., 2017) and breast cancers (Bae et al., 2017). Our findings support the notion that FLRT2 functions as a tumor suppressor in NSCLC. Moreover, we found that FLRT2 expression was significantly lower in NSCLC cells and even lower in NSCLC stem cells, indicating that FLRT2 may suppress cancer cell stemness in NSCLC. Tumor cell stemness is usually manifested by the upregulation of OCT4, CD133, and CD44, which were identified as key factors in maintaining stem cell properties, such as self-renewal, tumorigenesis, and drug resistance (Walcher et al., 2020; Zhou et al., 2023). In line with these findings, we found that FLRT2 upregulation significantly reduced the ability of NSCLC stem cells to form tumor spheres by decreasing the abundance of stemness-related genes, including OTC4, CD44, and CD133.

Additionally, our *in vivo* experiments demonstrated that upregulation of FLRT2 inhibited the tumorigenesis of NSCLC stem cells. Overall, our findings demonstrated that FLRT2 was downregulated in NSCLC tissues and cancer cells, and upregulation of FLRT2 inhibited the stemness and tumorigenesis of NSCLC^CSC^. As a newly defined tumor suppressor gene in NSCLC, FLRT2 might be a promising target for diagnosis and treatment of NSCLC.

As an E3 ligase, NEDD4 modulates protein degradation ubiquitination (Xu et al., 2022). In NSCLC tumors, NEDD4 was upregulated and promoted cancer cell tumorigenesis (Ye et al., 2014) and drug resistance (Sun et al., 2017; Shao et al., 2018). NEDD4 promotes NSCLC by degrading PTEN (Amodio et al., 2010; Sun et al., 2017), HMGCL (Zhong et al., 2023), and MEKK5 (Sun A. et al., 2021) proteins. NEDD4 also interacted with the EGFR signaling complex, increasing downstream cathepsin B secretion and NSCLC migration (Shao et al., 2018). In this study, we demonstrated that NEDD4 mediated the ubiquitination of FLRT2 and facilitated FLRT2 degradation, which inhibited NSCLC cell stemness. The Cignal Reporter Assay Kit was employed to explore the potential molecular mechanism(s) of the NEDD4/FLRT2 axis in NSCLC cell stemness.The stemness-related signaling pathways, such as HIF-1α (Gu et al., 2021), NF-kB (Xu et al., 2024), and TCF/LEF (Katoh and Katoh, 2022) were identified as potential downstream pathways of this axis. Overall, NEDD4 mitigated FLRT2 effects on NSCLC stemness by mediating FLRT2 ubiquitination and degradation. These findings suggest that the NEDD4-FLRT2 axis is a promising target for NSCLC treatment.

## Data Availability

The original contributions presented in the study are included in the article/Supplementary Material, further inquiries can be directed to the corresponding authors.

## Supplementary material

SUPPLEMENTARY FIGURE S1
**(A)** GEPIA2 data depicts that FLRT2 has non-significant downregulation in lung adenocarcinoma and lung squamous cell carcinoma. **(B)** Bioinformatic analysis revealed that FLRT2 expression negatively correlated with NSCLC cell stemness.

SUPPLEMENTARY FIGURE S2 Using the Unibrowser website to predict the target protein that may be involved in the ubiquitination of FLRT2.

SUPPLEMENTARY FIGURE S3 Cignal Reporter Assay Kit analyzes potential downstream molecular signals of the NEDD4/FLRT2 axis. All data are expressed as mean ± SEM of three independent experiments. **p* < 0.05, ***p* < 0.01, and ****p* < 0.001 assessed via a two-tailed t-test; ns: non-significant.
