# Supplementary figures and images for: The NEDD4/FLRT2 axis regulates NSCLC cell stemness
Source: Front Pharmacol. 2024 Oct 9;15:1459978. doi: 10.3389/fphar.2024.1459978 (PMC11496253; doi:10.3389/fphar.2024.1459978)

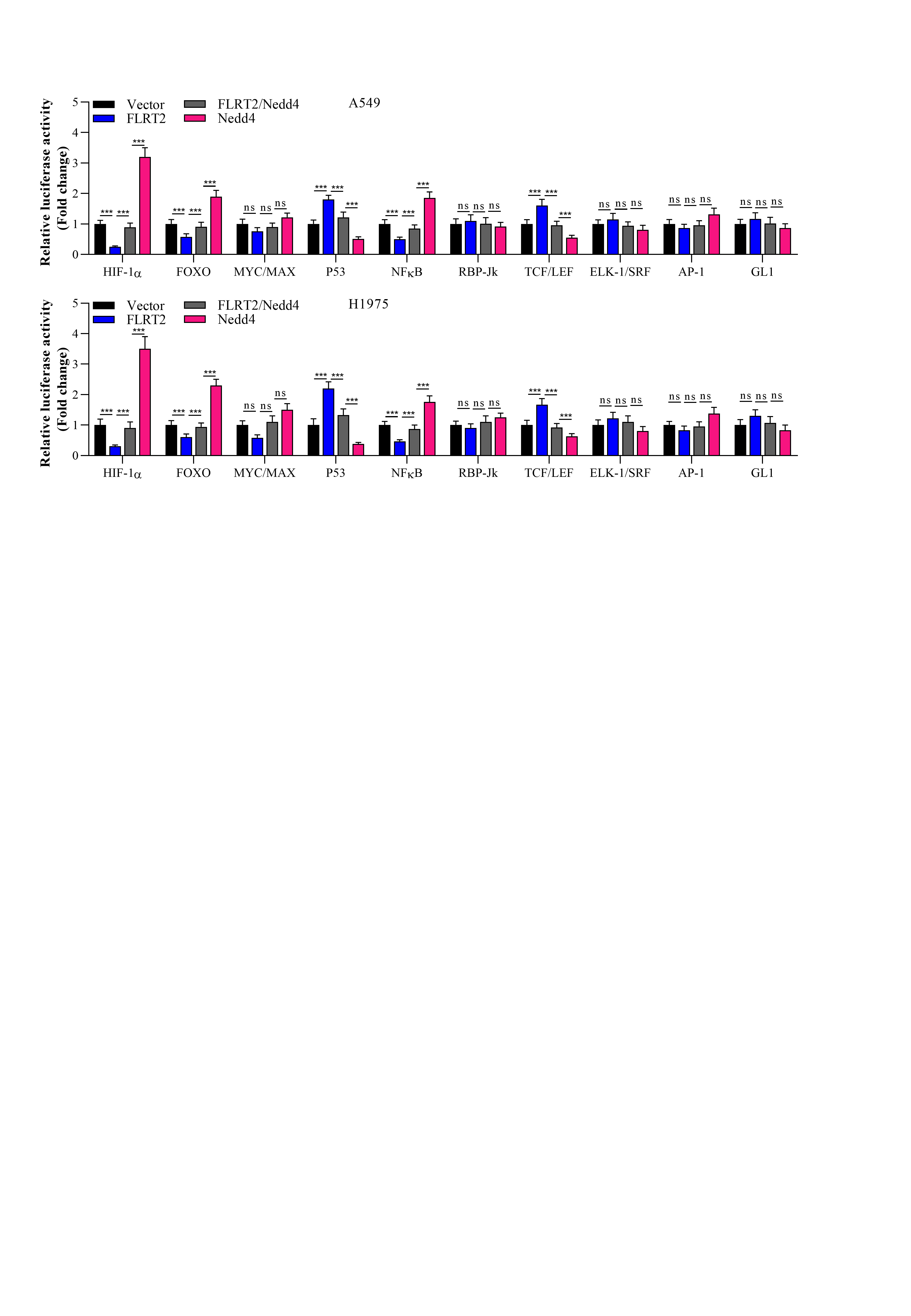

Supplement: Supplementary file 1 [file Image3.TIF]

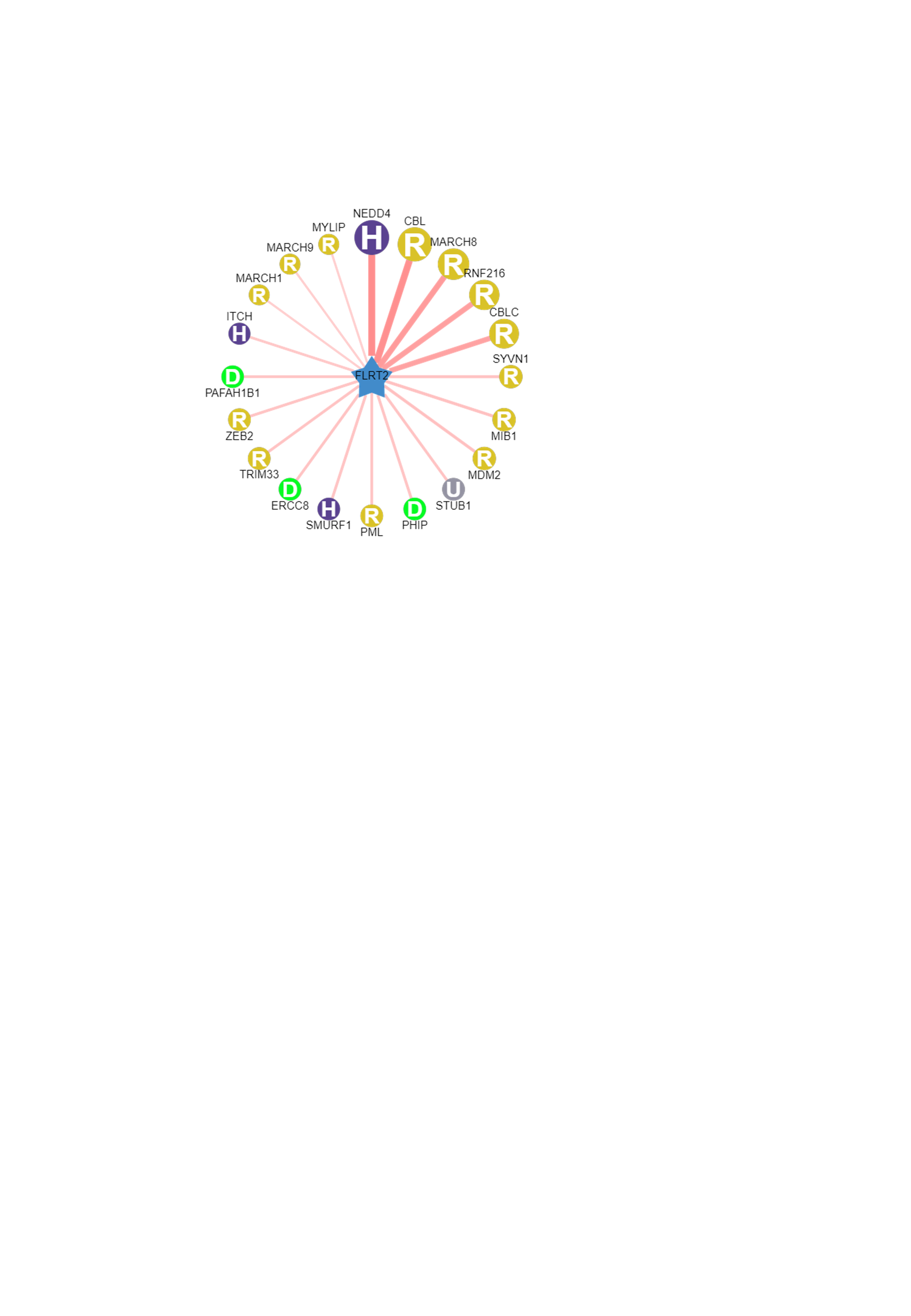

Supplement: Supplementary file 2 [file Image2.TIF]

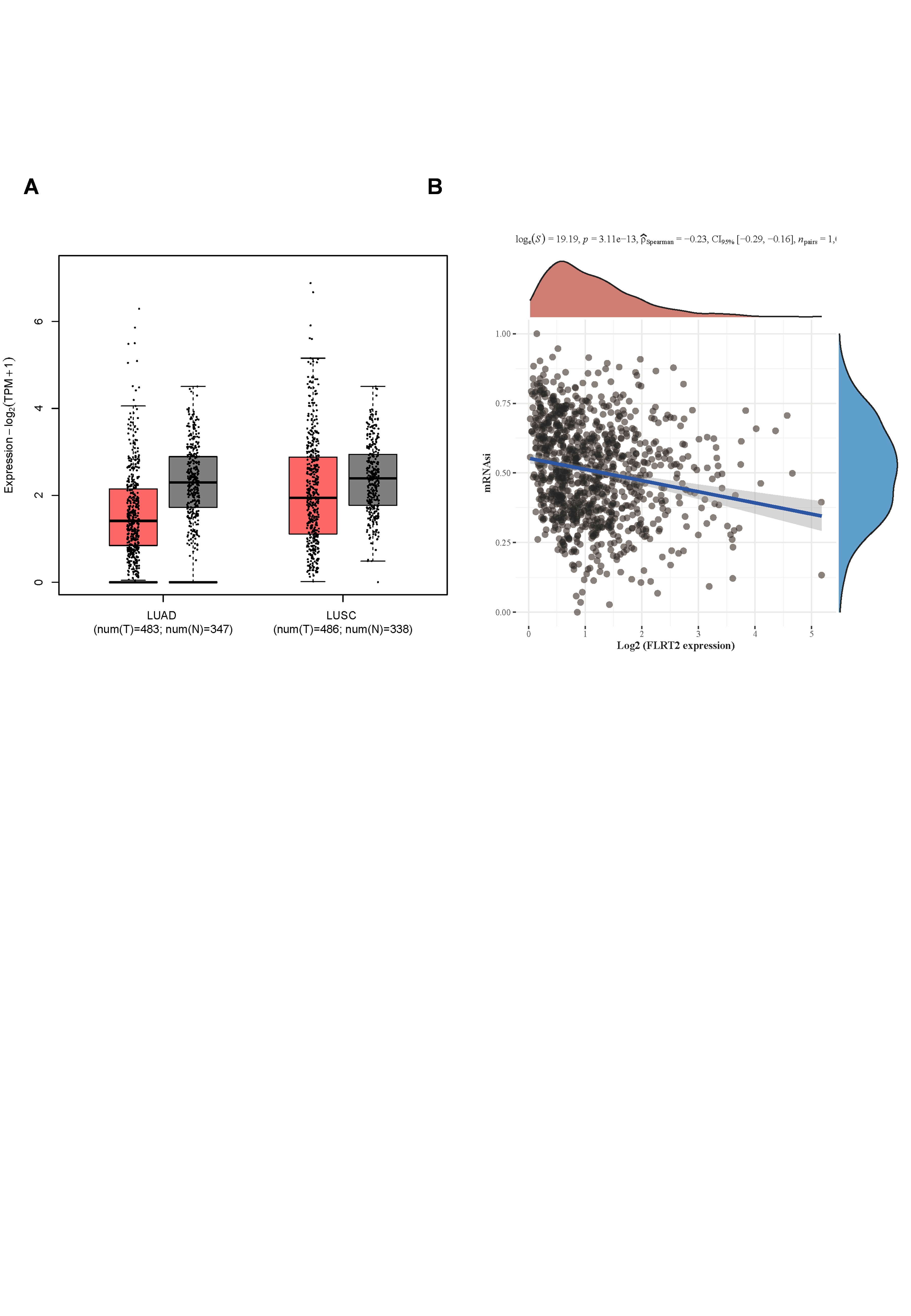

Supplement: Supplementary file 3 [file Image1.TIF]
